# Streptavidin functionalized hyaluronic acid hydrogels for controlled and customizable drug delivery

**DOI:** 10.1016/j.actbio.2026.07.015

**Published:** 2026-07-11

**Authors:** Emily E. Sharp, Lorielle Laforest, Yuqi Zhang, Karen L. Xu, Zhiliang Cheng, Jason A. Burdick, Sarah E. Gullbrand, Robert L. Mauck

**Affiliations:** a Department of Bioengineering, University of Pennsylvania, 210 S. 33^rd^ Street, 240 Skirkanich Hall, Philadelphia, PA 19104, USA; b McKay Orthopaedic Research Laboratory, Department of Orthopaedic Surgery, University of Pennsylvania, 3450 Hamilton Walk, Stemmler Hall, Philadelphia, PA 19104, USA; c CReATE Motion Center and Translational Musculoskeletal Research Center, Corporal Michael J. Crescenz VA Medical Center, 3900 Woodland Avenue, Philadelphia,PA 19104, USA; d BioFrontiers Institute and Department of Chemical and Biological Engineering, University of Colorado, 3415 Colorado Avenue, Boulder, CO 80303, USA; e Department of Physical Medicine and Rehabilitation, University of Pennsylvania, 1800 Lombard Street, Philadelphia, PA 19146, USA

**Keywords:** Biotin, MMP degradable, Liposome delivery, Subcutaneous implantation

## Abstract

Hyaluronic acid (HA)-based hydrogels have emerged as promising materials for tissue repair given their ability to rapidly gel *in situ* to precisely fill tissue defects, their mechanical tunability and customizable degradation kinetics, and their capacity to deliver therapeutic agents to the site of injury. HA hydrogels can also be used to encapsulate therapeutics for local delivery; however, release through diffusion may be too rapid to achieve a therapeutic window for many applications. To overcome this limitation, we functionalized norbornene-modified HA (NorHA) hydrogels with streptavidin using thiol-ene click chemistry, enabling efficient tethering of biotin-modified agents (i.e., fluorophores, liposomes) to the hydrogel. We first confirmed that streptavidin functionalization increased the retention of encapsulated agents both *in vitro* and *in vivo* and did not impact hydrogel properties (including rapid gelation or mechanical tunability). Next, we incorporated matrix metalloproteinase (MMP) degradable crosslinks to further tune hydrogel degradation, permitting increased cellular invasion *in vivo* without disrupting retention and delivery of biotin-modified fluorophores. Lastly, streptavidin functionalized hydrogels were used to encapsulate and control the release of liposomes, with customizable release of model therapeutics within the liposome core and membrane. Overall, these findings support a promising material to control the release of therapeutics that could be useful in a variety of biomedical applications.

## Statement of Significance

Soft tissue injuries are ubiquitous and a significant cause of disability in society. Functionalized hydrogels enable controlled delivery of therapeutics to promote tissue repair. In this study we demonstrated that streptavidin functionalization of hyaluronic acid-based hydrogels successfully tethers biotin-modified agents, prolonging their localized delivery. Functionalization did not impact hydrogel gelation, mechanical tunability, or the ability to tailor hydrogel degradation. Further, the tethering of biotin-modified liposome nanocarriers enabled staged delivery of multiple agents. Altogether, our results highlight streptavidin functionalized hydrogels as a promising platform to promote endogenous healing and tissue regeneration through controlled spatiotemporal delivery.

## 1. Introduction

In the United States, musculoskeletal injuries and disorders affect 1 in 3 people [1,2] and are the second-highest ranked cause of non-fatal disability [3]. A number of promising regenerative strategies have emerged to address the diverse conditions affecting joints, muscles, and connective tissues, such as orthobiologics [4], cell therapies [5], and reparative biomaterials [6–8]. In many approaches, biomaterials are utilized to provide structural support at an injury site and to localize cell and biologic therapies. Hydrogels (water swollen crosslinked networks) are particularly advantageous for these applications given their ability to mimic properties of native tissue microenvironments [9], their ease of application (i.e., injectable, minimally invasive) [10], and their customizability [11]. While hydrogels can be formed from numerous synthetic and natural polymer backbones, natural systems are advantageous based on their biocompatibility and potential for cell-mediated degradation.

One such widely-used natural polymer, hyaluronic acid (HA), is a long-chain glycosaminoglycan found in large amounts in the extracellular matrix (ECM) of musculoskeletal tissues, especially in the meniscus, cartilage, and intervertebral disc [12]. Chemical modifications to HA can be made through single bond formation to one of the three main functional groups (hydroxyl, carboxyl, or N-acetyl) to enable formation of stable HA hydrogels [13]. Common modifications include methacrylate, acrylate, norbornene, guest-host chemistry, maleate, thiol, tyramine, and sulphate [13,14]. These modifications provide distinct methods for customizing hydrogel biophysical properties, such as stiffness, degradation, injectability, and self-healing capabilities. This in turn enables one to tailor hydrogel properties to fulfill design criteria for a variety of biomedical applications. Within the field of musculoskeletal soft tissue regeneration, a variety of HA-modified hydrogels [15–19] have been investigated, particularly with methacrylate-modified HA (MeHA) [20–24] and norbornene-modified HA (NorHA) [25–29]. Both MeHA and NorHA hydrogels can be formed via visible and UV light mediated crosslinking, making them advantageous for applications requiring *in situ* gelation. The physical properties of these hydrogels can also be readily tuned by varying the polymer concentration, degree of modification, and crosslink density [30]. Fibrous [31] and granular [32] HA-based hydrogels are easily fabricated using a number of fabrication methods, which alters the hydrogel structure and mediates cellular interactions.

Beyond their ability to fill defects and deliver cells, hydrogels can also encapsulate and deliver therapeutics to influence local cell behavior and stimulate repair, with the hydrogel mesh size and degradation rate (along with the molecular weight of the therapeutic) regulating release profiles [8]. Encapsulated therapeutics, however, typically release by free diffusion and the total release occurs prior to complete hydrogel degradation [33]. Release through diffusion may be too rapid to achieve a therapeutic effect, presenting a notable limitation for many applications. In applications where sustained release is desirable, researchers have employed tethering, where therapeutics are bound to the polymer network, and affinity binding, where therapeutics are retained within the hydrogel via molecular interactions [34,35]. HA modifications can be used to enable tethering and affinity binding of a desired agent directly with the HA backbone. An example of tethering is the reaction of thiol-modified therapeutics with the norbornene residues of NorHA hydrogels [36,37]. In this approach, the same chemistry is used for both tethering and hydrogel crosslinking, meaning that therapeutic release is controlled by hydrogel degradation unless another mechanism for release is introduced. Alternatively, the hydrogel backbone itself can be functionalized, where a secondary reaction binds an agent, which has been investigated for the attachment of labelling dyes [38,39] and cell adhesion peptides [40,41]. This HA backbone functionalization provides an alternate methods to control therapeutic attachment and prolonged release.

Here, we functionalized NorHA hydrogels to enable customizable, spatiotemporal release of multiple therapeutics, where both the location and timing of therapeutic release can be controlled. The injectable nature of HA hydrogels facilitates easy administration of the hydrogel, enabling localized delivery. To control therapeutic release, we utilized thiol-based linkers to tether streptavidin to the HA backbone (via thiolene reactions), enabling secondary reactions between streptavidin and desired biotin-modified agents (e.g., fluorophores, biologics, nanocarriers) (Fig. 1A). This biotin-streptavidin binding is well established and has been used in a variety of biomedical applications given its high specificity and strong binding coefficient [42]. Unlike other tethering methods that also use thiol-ene chemistry, which attach therapeutics via one-to-one additions, our streptavidin functionalized hydrogels have an increased number of binding sites due to the tetrameric structure of streptavidin. This increases delivery potential and enables researchers to more easily customize therapeutic delivery without requiring a complex synthesis or fabrication process. Specifically, we incorporated thiol-streptavidin (TS) into a NorHA hydrogel precursor solution such that, upon UV exposure, hydrogel crosslinking and streptavidin functionalization occur simultaneously. With this, biotin-modified agents can form non-covalent bonds with streptavidin to tether the agent to the hydrogel for controlled delivery. We hypothesized that streptavidin functionalization would not impact hydrogel crosslinking, material properties, or tunability, as it only reacts with a very small fraction of the norbornene groups, while enabling strong secondary biotin-streptavidin reactions that endure *in vivo* to prolong the time course of therapeutic delivery.

## 2. Methods

### 2.1. Hydrogel fabrication and functionalization

Norbornene-modified hyaluronic acid (NorHA) was synthesized using Boc_2_O (di-tert-butyl decarbonate) coupling chemistry of norbornene to HA (65 kDa, Lifecore) and was collected in high yield following dialysis [30]. The quality and percentage of HA repeat units modified with pendant norbornenes was determined via ^1^H NMR [30]. All experiments were performed using NorHA that was modified at 20% (Fig. S.1). This modification was chosen because it is easily and reproducibly synthesized, and it provides a sufficient number of pendant norbornenes for both hydrogel formation and functionalization. The percent modification along with desired NorHA concentration will dictate the exact molar concentrations for crosslinking and functionalization. Exact hydrogel formulations for all hydrogels used in this study were provided in Table S.1. Hydrogels were formed by first dissolving NorHA in PBS to reach the desired concentration. Unless otherwise specified, hydrogels were formed with either 3 or 5 wt% NorHA. This crosslinker concentration used would theoretically consume 50% of the available norbornenes during reaction, which allowed stable hydrogel formation while reserving the remaining half of the norbornenes for functionalization. Crosslinking levels could be further increased to increase hydrogel stiffness, so long as the number of thiols does not exceed the number of norbornenes, which leads to hydrogel softening [30]. Crosslinking was accomplished via dithiothreitol (DTT) and/or di-thiolated MMP-degradable (MD) crosslinkers (GenScript, GCNSVPMSMRGGSNCG) [22]. Irgacure 2959 (0.5 mg/mL) was used as the photo-initiator. Individual gels were formed by placing 50 μL of precursor solution into a cylindrical mold (4 mm diameter, 5 mm height) and hydrogels were crosslinked via a thiol-ene reaction using UV light (365 nm, 10 mW/cm^2^, 2 min).

Hydrogel functionalization was accomplished by incorporating thiol-streptavidin (TS) (ProteinMods, 2.8 thiols/tetramer) directly into the hydrogel precursor solution prior to gel formation. Thus, both the crosslinks and the TS undergo thiol-ene reactions simultaneously upon UV exposure. Unless otherwise specified, 1% of the norbornenes attached to the backbone were functionalization with TS.

### 2.2. Secondary reaction validation

Hydrogels were fabricated with varying levels of TS functionalization (0, 0.5, 1, and 2.5%). During fabrication, biotin-modified Cy5 was included in the precursor solution at a concentration of 30 μM as a model therapeutic. Hydrogels were washed in PBS supplemented with 10% FBS and 1% PSF on an orbital shaker at 37° C for up to 28 days. Washing medium was collected and replaced every 2–3 days. After 28 days, hydrogels were digested in 100 U/mL hyaluronidase. Media was frozen, lyophilized, and resuspended in a smaller volume of PBS for analysis (Synergy H1 Microplate; 650 nm excitation, 680 nm emission). Release from a total of n=6 hydrogels/group was assayed.

### 2.3. Mechanical characterization

Unconfined stress relaxation protocols were performed on an Instron 5948. Hydrogels were kept hydrated in PBS for the duration of testing. Hydrogels were compressed to 10% strain at a rate of 0.05% per second and this was followed by a 1000 s hold. This was repeated two additional times for a total of three cycles. The changes in equilibrium stress during the second and third cycles were averaged to determine the equilibrium modulus [43]. A total of n=6 hydrogels/group was used for mechanical analysis of the bulk hydrogels. Additional rheological characterization was performed on a HR30 TA Rheometer with a 20 mm, 2° cone plate geometry. Storage and loss moduli were continuously monitored while the hydrogel precursor solution was exposed to UV light (10 mW/cm^2^) for two minutes by applying 1% strain at a 1 Hz frequency. Gel point was determined by identifying the intersection of the storage and loss modulus curves. A total of n=4 hydrogels/group was used for this rheologic analysis.

### 2.4. In vivo characterization

NorHA was sterilized for 60 min via irradiation by a germicidal lamp, and all other reagents involved in hydrogel fabrication were sterile filtered (0.22 μm). 0% and 1% TS functionalized hydrogels were fabricated with varying degrees of MMP-degradable crosslinks (0, 50, 100%). Hydrogels were crosslinked to 50% of available norbornene residues, with DTT provided to form additional crosslinks to achieve this level of total crosslinks. Thiolated-RGD was incorporated into the hydrogel precursor solution (1 mg/mL) to promote cell adhesion and infiltration. A third of the hydrogels also included 30 μM biotin-modified Cy5 to confirm the biotin-streptavidin secondary reaction and persistence *in vivo*.

Hydrogels were implanted subcutaneously in 6-week-old, male Sprague-Dawley rats (Charles River) using aseptic techniques. Rats were anesthetized with isoflurane, and bupivacaine (0.25%) and meloxicam (2 mg/mL) were delivered for analgesia via subcutaneous injection. Six dorsal pockets were opened using sharp and blunt dissection (Fig. 2A). 0% TS hydrogels were implanted in the left pockets, and 1% TS hydrogels were implanted in the right pockets. 0% MD hydrogels were implanted in the proximal pockets, 50% MD hydrogels were implanted in the medial pockets, and 100% MD hydrogels were implanted in the distal pockets. Only one hydrogel was inserted into each pocket, and the incision was closed using surgical staples. Rats were returned to normal cage activity after the procedure and staples were removed after 7 days. Rats were euthanized on either day 7 or day 14 post-implantation. Implanted hydrogels were used to monitor the secondary reaction between hydrogel and encapsulated biotin-modified Cy5 (described above), hydrogel degradation via stress relaxation (described above), and cell infiltration via histologic evaluation with Hematoxylin and Eosin (H&E) staining and 4′,6-diamidino-2-phenylindole (DAPI) nuclear staining. Quantification of cell infiltration was performed in ImageJ. Images were all manually thresholded to reduce the background signal from the hydrogel and isolate infiltrating cells. Areas of hydrogel identifiable within the fibrous capsule were manually outlined and fluorescence intensity and area were measured. Fluorescence from non-cellular artifacts or hydrogel were subtracted. A portion of the samples fabricated on the day of surgery were immediately processed for each outcome and served as pre-implantation, day 0 controls. A total of 24 rats were used to reach an n=4 per hydrogel group for each outcome measure at each timepoint. This study was approved by the Institutional Animal Care and Use Committee of the University of Pennsylvania (IACUC #806,580) and was conducted in compliance with the NIH Guide for Care and Use of Laboratory Animals.

### 2.5. Nanocarrier fabrication and secondary attachment

A lipid stock solution in chloroform was prepared by combining 4 mg of 1,2-dipalmitoyl-sn-glycero-3-phosphocholine (DPPC), 0.1 mg of 1,2-dioleoyl-sn-glycero-3-phosphoethanolamine-N-(Cyanine 5, Cy5) (18:1 Cyanine 5 PE), and 1 mg of 1,2-distearoyl-sn-glycero-3-phosphoethanolamine-N-[biotinyl(polyethylene glycol)-2000] (DSPE-PEG(2000) Biotin) (Avanti Polar Lipids, Inc.). Control liposomes (i.e., without Biotin) were prepared using the same amount of lipids but without DSPE-PEG (2000) Biotin. The lipid stock solution was dried under nitrogen in a glass vial for 10 min and further dried under vacuum for at least 4 hours to ensure complete removal of chloroform. The dried lipid film was then reconstituted with 1 mL of FITC-labeled dextran (10 mg/mL; MW 6000 Da, Creative PEGWorks) in 10 mM phosphate buffer (pH 7.4). After incubation in a 45 °C water bath for 30 min, the liposome samples underwent ten freeze–thaw–vortex cycles using liquid nitrogen and warm water (45 C), respectively. The resulting suspension was extruded 21 times through a 100-nm Nuclepore polycarbonate membrane using a stainless-steel extruder (Avanti Polar Lipids, Inc.). Non-encapsulated FITC-labeled dextran was removed by size-exclusion chromatography on a Sepharose CL-4B column (Sigma). The size distribution of purified liposomes was measured by dynamic light scattering (DLS) using a Zetasizer Nano S (Malvern Instruments), and fluorophore concentrations were quantified spectrophotometrically with a SPEX FluoroMax-3 spectrofluorometer.

Biotin-modified and non-modified liposomes (30 μM) were encapsulated in 0% and 1% TS functionalized hydrogels with 30% MD crosslinks. Hydrogels were incubated overnight in a washing medium consisting of PBS supplemented with 10% FBS and 1% PSF on an orbital shaker at 37° C. The washing medium was collected and replaced with 50 U/mL collagenase for two days to degrade the MD crosslinks and provide additional space for non-attached liposomes to be released. The digestion medium was collected and replaced with washing medium (PBS supplemented with 10% FBS and 1% PSF) for the remaining 12 days of the experiment. Washing medium was collected and replaced every 2–3 days. Medium was frozen, lyophilized, and resuspended in a smaller volume of PBS for analysis (described above). A total of n=11 hydrogels/group was used for these assays.

### 2.6. Statistical analysis

Statistical analyses were conducted in Prism (Graph Pad Software Inc., La Jolla, CA). Significance was defined as p<0.05. All data are shown as mean ± standard deviation, unless otherwise indicated. Outliers were identified using a ROUT test. Normality was assessed using Shapiro-Wilk tests. A Two-Way Repeated Measures Analysis of Variance (ANOVA) with Tukey’s multiple comparisons test was used to examine statistical differences between groups for fluorescence attachment to TS functionalized hydrogels *in vitro*. A Mann-Whitney non-parametric t-test was used to determine statistical significance in gel point between 0% and 1% TS hydrogels. Two-Way ANOVA with Sidak’s multiple comparisons were used to determine statistical differences *in vitro* and *in vivo* equilibrium modulus, normalized equilibrium modulus of MD hydrogels, and fluorescence attachment to MD hydrogels after subcutaneous implantation. One-Way ANOVA with Tukey’s multiple comparisons was used to examine the statistical difference between fluorescence attachment to implanted MD hydrogels and pre-implanted controls. A Kruskal-Wallis non-parametric test with multiple comparisons was used to evaluate statistical differences in cell infiltration. A Two-Way ANOVA with Tukey’s multiple comparisons test was used to evaluate biotin-modified liposome retention and payload release. Unpaired, parametric t-tests with Welch’s correction were used to examine statistical differences in fluorescence attachment to 0% MD hydrogels post-implantation as well as liposome retention and payload release from liposomes with and without biotin modification. Information on each of the associated statistical tests is provided in the figure legends.

## 3. Results

### 3.1. Streptavidin functionalization permits secondary reactions without sacrificing hydrogel tunability

NorHA hydrogels were successfully functionalized by incorporating thiol-streptavidin (TS) into the hydrogel precursor solution prior to UV exposure (Fig. 1A). When biotin-modified fluorophores were encapsulated within hydrogels of varying levels of TS functionalization, release kinetics depended on hydrogel functionalization. In 0% TS (non-functionalized) hydrogels, nearly all the biotin-modified fluorophore was released within 1 day, and the remainder was released within 3 days (Fig. S.2). Conversely, hydrogels functionalized with even low levels of TS retained the biotin-modified fluorophore for over 21 days (Fig. 1C). This retention of fluorophore was visually apparent at each timepoint (Fig. 1B), and quantification of release revealed no significant differences between the 0.5%, 1%, and 2.5% TS functionalized hydrogels at any timepoint, likely due to the tetrameric structure of streptavidin enabling binding of multiple biotin-modified fluorophores to a single unit of TS (Fig. 1C, Fig. S.2). Given that there were no significant differences between TS functionalized hydrogels, 1% TS hydrogels were utilized for all further experiments. This allowed for sufficient attachment of biotin-modified agents while preserving resources (TS quantity).

Given that the thiol-streptavidin (TS) is a large molecule (MW > 60 kDa), we next evaluated whether inclusion of this molecule altered gel formation or final hydrogel properties. 1% TS functionalization did not impact the equilibrium modulus of bulk hydrogels compared to non-functionalized hydrogels. Functionalization also did not impact the mechanical tunability of the hydrogel, as demonstrated by increased equilibrium modulus with increased NorHA concentration (Fig. 1D). These hydrogels are stable after formation, having comparable equilibrium moduli after 6 months of storage in PBS at 4 °C for both 0% and 1% TS hydrogels (Fig. S3). There were also no significant differences in gel point between functionalized and non-functionalized hydrogels (Fig. 1E). There were also no significant differences in storage modulus after gelation (Fig. S.4). These data indicate that TS functionalization enables biotin-mediated retention of molecules while preserving NorHA hydrogel tunability.

### 3.2. Streptavidin functionalization enables retention of biotin-modified therapeutics in vivo

To evaluate *in vivo* performance of these functionalized hydrogels, they were implanted subcutaneously in rats for 7 and 14 days (Fig. 2A). When loaded with a biotin-fluorophore, 1% TS functionalized hydrogels had significantly higher fluorescence intensity compared to non-functionalized hydrogels after 14 days of subcutaneous implantation (Fig. 2B). Notably, there was no significant difference between the fluorescence intensity measurements for 1% TS functionalized hydrogels implanted for 14 days and those of non-implanted hydrogel controls that were fabricated at the time of implantation (grey dashed line). Fluorophore retention was visually evident at 7 and 14 days (Fig. 2C).

The differences in release behavior observed were not related to changes in mechanical properties, given that there were no significant changes in equilibrium modulus in 1% functionalized TS hydrogels between 0 and 14 days of implantation (Fig. 2D). While there was a significant decrease in stiffness in non-functionalized hydrogels by day 14 (42% reduction), no significant differences were detected between 0% and 1% TS functionalized hydrogels at any timepoint. H&E histology also showed minimal hydrogel fragmentation at day 7 and only mild fragmentation at day 14 (Fig. 2E). There were no signs of cell infiltration into the hydrogel at either timepoint, regardless of TS functionalization.

### 3.3. MMP-degradable crosslinks expedite cell infiltration while retaining therapeutics

Cellular infiltration is important to initiate nascent tissue deposition and remodeling following hydrogel repair of a tissue. Given our observation of only moderate changes in mechanical properties by day 14 and little cellular invasion, we next incorporated MMP-degradable (MD) crosslinks (Fig. 3A) to hasten hydrogel degradation and boost cellular infiltration. To verify successful introduction of MD crosslinks, we measured swelling ratio before and after incubation of hydrogels in a collagenase solution, across a range of MD crosslinking percentages (Fig. S5A). As expected, there was a slight (but non-significant) increase in swelling ratio after MMP degradation and hydrogels with 100% MD crosslinks completely degraded. We also confirmed that the incorporation of MD crosslinks did not adversely impact biotin-streptavidin secondary reactions (Fig. S5B). Immediately following fabrication, there were no significant differences in equilibrium modulus with MD crosslink incorporation into 1% TS hydrogels (Fig. 3B, Fig. S6). After 7 days of implantation of 1% TS hydrogels, there was a 52% reduction in 50% MD hydrogels compared to 0% MD hydrogels (11 v. 23 kPa) and an 84% reduction in 100% MD hydrogels compared to 0% MD hydrogels (4 v. 23 kPa). After 14 days of implantation, there was a 100% reduction in 50% MD hydrogels (0 v. 14 kPa) and a 79% reduction in 100% MD hydrogels (3 v. 14 kPa). These reductions were all statistically significant, and there were no significant differences between 50% and 100% MD hydrogels at either timepoint. 0% TS hydrogels behaved similarly to 1% TS hydrogels during implantation (Fig. S7).

Consistent with this loss in mechanical properties, H&E staining of implanted hydrogels showed some fragmentation after 7 days and marked changes in the 50% and 100% MD groups at 14 days of implantation (Fig. 3D). Hydrogels comprised of entirely MD crosslinks (100%) showed the most fragmentation at each timepoint, while hydrogels with half MD crosslinks (50%) showed a lesser degree of fragmentation. As previously noted, hydrogels lacking MD crosslinks (0%) showed no changes. Hydrogels containing MD crosslinks showed regions of cell infiltration and nascent matrix deposition along the disrupted interfaces (Fig. 3E), with significant differences in cell infiltration after 14 days (Fig. 3C, Fig. S.10). Non-functionalized hydrogels showed similar behavior (Fig. S.8, Fig. S.9).

We also monitored retention of biotin-modified fluorophores in these MD hydrogels with subcutaneous implantation (Fig. 4A). Retention of the fluorophore did not change as a function of MMP-degradable crosslinks, with TS functionalized hydrogels retaining the full complement of encapsulated biotin-modified fluorophore over 14 days of implantation (Fig. 4B). Importantly, this retention occurred despite the physical breakdown and cellular invasion of the hydrogel (Fig. 4C). As with 0% MD hydrogels, TS functionalized hydrogels had significantly higher fluorescence intensity compared to non-functionalized hydrogels for both 50% and 100% MD hydrogels (Fig. 4B). There was also no significant difference in fluorescence intensity for TS functionalized hydrogels compared to their pre-implanted hydrogel controls. All additional data collected from the subcutaneous implantation experiments are shown in Fig. S11.

### 3.4. Streptavidin functionalization enables staged release via localization of biotin-modified liposomes

To extend these studies to consider additional types of biotin-modified therapeutics, we explored how liposomes could be incorporated into the system. Liposomes fabricated with chemically conjugated Cy5 in the outer shell and FITC-labeled dextran in the inner core were biotin-modified and encapsulated in 0% and 1% TS functionalized hydrogels containing 30% MD crosslinks (Fig. 5A). Over 14 days, the FITC dye was steadily released from both hydrogel groups, with FITC releasing slightly slower from 1% TS functionalized hydrogels (Fig. 5B). Slightly less (3% reduction) FITC was released from biotin-modified liposomes compared to non-modified liposomes encapsulated in 1% TS functionalized hydrogels (Fig. 5C). The Cy5 fluorophore, either from unbound liposomes or fragmented liposomes, began releasing from the hydrogels after MD crosslink degradation on day 3 (Fig. 5D). Notably, 1% TS functionalized hydrogels retained Cy5 signal to a greater extent than non-functionalized hydrogels. Additionally, after 14 days of incubation, nearly twice as much Cy5 was retained from the biotin-modified liposomes compared to non-modified liposomes that were encapsulated in 1% TS functionalized hydrogels (Fig. 5E). These data indicate that, despite the physical breakdown of the MD crosslinks in TS functionalized hydrogels, biotin modified therapeutics can be retained over long time periods, during which cellular infiltration occurs.

## 4. Discussion

Functionalized hydrogels that can tether and release therapeutics locally over a wide time window are of great interest for musculoskeletal tissue repair. Prior studies have used this approach to tether growth factors and growth factor mimicking peptides [44,45], for a diverse array of applications, including bone [44,46,47], cartilage [45,48,49], muscle, meniscus, and disc repair [50–54]. Here, we built on this platform by functionalizing NorHA hydrogels with thiol-streptavidin (TS). Within the cancer research field, researchers have modified HA to employ biotin-streptavidin chemistry for drug delivery purposes, using intricate photocage methods to immobilize streptavidin-modified growth factors in HA hydrogels [55] and coating mesoporous silica nanoparticles modified with streptavidin in biotin-modified HA to extend delivery of an anti-cancer model drug [56]. In contrast to these elaborate methods for functionalization, TS functionalization is achieved in our work by incorporating all reagents directly into the hydrogel precursor solution, creating a simple and effective method to successfully tether biotin-modified agents to the hydrogel backbone. We confirmed that, despite its large size, TS functionalization did not compromise hydrogel integrity or mechanical tunability. *In vivo*, TS functionalized hydrogels retained a biotin-modified model drug, maintaining as much as pre-implanted controls and confirming successful tethering. TS functionalized and non-functionalized hydrogels exhibited slow degradation kinetics and showed no signs of cell infiltration, despite the presence of adhesive RGD moieties. Depending on the intended application, a lack of cell infiltration into the hydrogel may have positive or negative implications. Within the context of cancer immunotherapies, for example, the lack of cell infiltration may help maintain the structural integrity of the hydrogel and ensure that any therapeutics are not rapidly cleared [57]. In musculoskeletal tissue regeneration applications, however, cell infiltration is desirable for long term functional repair of the injured tissue [58,59].

To improve cell infiltration, we replaced a portion of the dithiol crosslinks with MMP-degradable (MD) crosslinks to tune hydrogel degradation, as is done routinely in the field [60–64]. As anticipated, introducing MD crosslinks increased the rate of hydrogel degradation and fragmentation. This created pathways for cells to infiltrate the fragmented hydrogel following *in vivo* implantation and deposit nascent matrix. Thus, the degree of MD crosslinks is another variable that can be customized to control hydrogel mechanics and degradation as well as influence cellular engagement with the hydrogel.

Importantly, the incorporation of MD crosslinks and associated changes to hydrogel degradation did not interfere with the tethering of a biotin-modified model drug via TS functionalization. As observed in 0% MD hydrogels, TS functionalized hydrogels with 50% and 100% MD crosslinks retained as much fluorophore as pre-implanted controls. These results highlight the strength and durability of the biotinstreptavidin bonds, persisting despite substantial hydrogel fragmentation. These results also confirm that TS reacted with the pendant norbornenes and was not solely encapsulated in the hydrogel matrix. That is, if the TS was simply trapped in the hydrogel, fluorescence intensity would have steadily decreased with hydrogel degradation. Instead, we observed stable retention of fluorescence intensity at each timepoint. Taken together, these findings demonstrate that we can successfully engineer degradation without altering the time course of localization of a therapeutic.

To demonstrate that this hydrogel system can also be used to deliver multiple therapeutics, we monitored the release of two fluorophores as model therapeutics from biotin-modified liposomes. Liposomes are lipid-based nanocarriers that can deliver both a hydrophobic therapeutic in its outer shell and a hydrophilic therapeutic in its inner core [65]. Hydrophobic drugs are often challenging to deliver via hydrogels due to their limited water solubility; however, liposomes overcome this limitation. We observed that biotin-modified liposomes incorporated into TS functionalized hydrogels steadily released a desired cargo (modeled by FITC) and prolonged the delivery of a secondary therapeutic contained within the outer shell (modeled by Cy5). FITC release kinetics were similar when liposomes were encapsulated in non-functionalized hydrogels, but Cy5 released faster from liposomes in non-functionalized hydrogels. Given the relatively large size of the FITC-dextran (MW=6 kDa), we interpret the steady release of FITC to indicate liposome breakdown. Thus, the retention of Cy5 suggests that portions of the biotin-modified liposome shell remain in the hydrogel due to successful tethering with streptavidin. These results indicate that biotin-modified liposomes could be utilized with TS functionalized hydrogels to achieve staged therapeutic delivery.

With respect to feasibility of clinical translation, this method of functionalization is easy to implement as it employs the same thiol-ene click chemistry reactions used to crosslink the hydrogel [66,67] and requires only one UV crosslinking step. This method also allows for easy customization of the degree of functionalization, as it does not depend on the reaction efficiency achieved during polymer synthesis. Our data demonstrates that the incorporation of low levels of TS is sufficient to significantly increase retention of a biotin-modified model drug, even after substantial hydrogel degradation. This is due to the tetrameric structure (i.e., four binding sites) of streptavidin. While researchers have attached peptides and other biologics to hydrogels via similar thiol-ene reactions [68–73], these reactions are generally one-to-one additions. Because of the increased number of binding sites, streptavidin functionalized hydrogels therefore have increased delivery potential compared to hydrogels where the therapeutic is bound directly to the hydrogel via thiol-ene reactions. Additionally, biotinylated proteins and nanocarriers are commercially available resources ready to be utilized with a compatible delivery platform, such as our TS functionalized hydrogels.

Despite the potential of this drug delivery platform, there are several limitations that should be considered for future applications. First, this method of crosslinking and functionalization is UV-mediated. Hydrogel formation by irradiation *in vivo* is currently limited to superficial depths (~0.5 mm at 365 nm - 500 nm) [74], which is sufficient for many (but not all) tissue repair applications. Second, we primarily characterize the release behavior of 1% TS hydrogels, but higher amounts of TS functionalization could be utilized to increase loading capacity. In future work, it would be useful to know how much functionalization is possible before compromising hydrogel integrity. Due to the low level of TS functionalization used in this study and the challenges associated with measuring streptavidin using NMR, we were unable to quantify and demonstrate TS functionalization beyond the validation experiments using biotin-modified fluorophores. Additionally, the aim of this study was to demonstrate spatiotemporal release from this hydrogel system and, thus, we did not disentangle the relative contributions of diffusion, affinity-mediated retention, and hydrogel degradation on the release kinetics. However, our results highlight that the release of biotin-modified therapeutics depends primarily on the total breakdown of the hydrogel due to the exceptionally high binding strength of biotin-streptavidin interactions. Although they are non-covalent in nature, biotin-streptavidin interactions outcompete the strength of the covalent thiol-norbornene bonds formed during crosslinking [75]. We demonstrated that introducing MMP degradable crosslinks enables a customized degradation rate to tailor the release. However, we did not explore the use of enzymatically degradable linkers on either the thiol-streptavidin or the biotin-modified agent, which could be used to release tethered molecules without altering the greater hydrogel network [71]. Lastly, we did not directly investigate streptavidin immunogenicity, which is a reported obstacle in cancer immunotherapy treatments [76–79]. While our histological results did not show adverse immune effects with TS functionalization, likely due to the relatively small amount of streptavidin in each hydrogel, future work employing increased levels of TS functionalization should monitor this carefully. Additional future directions include confirming the bioactivity of therapeutics delivered using this hydrogel system and examining their release kinetics as well as the delivery of multiple therapeutics at different timescales, such as by attaching multiple different drugs via biotin-streptavidin reactions as well as combining bulk encapsulation with prolonged delivery of another drug via biotin-streptavidin reactions.

## 5. Conclusion

HA functionalization has been used to enhance cell adhesion, direct cell behavior, control hydrogel degradation, and modulate immune responses [13]. Functionalization, however, is currently underutilized as a method to prolong therapeutic delivery. Here we demonstrate successful streptavidin functionalization of NorHA hydrogels via thiol-linkers, enabling localization and retention of a biotin-modified agent (e.g., fluorophores, biologics, nanocarriers, etc.) via secondary biotin-streptavidin interactions. This functionalization enables prolonged therapeutic delivery without sacrificing biocompatibility, altering gelation kinetics or mechanical tunability, or changing hydrogel degradation kinetics. These streptavidin functionalized hydrogels offer a promising platform to promote endogenous healing through precise spatiotemporal delivery of multiple therapeutics.

## Supplementary Material

supplemental materials

Supplementary material associated with this article can be found, in the online version, at doi:10.1016/j.actbio.2026.07.015.

## Figures and Tables

**Fig. 1.**
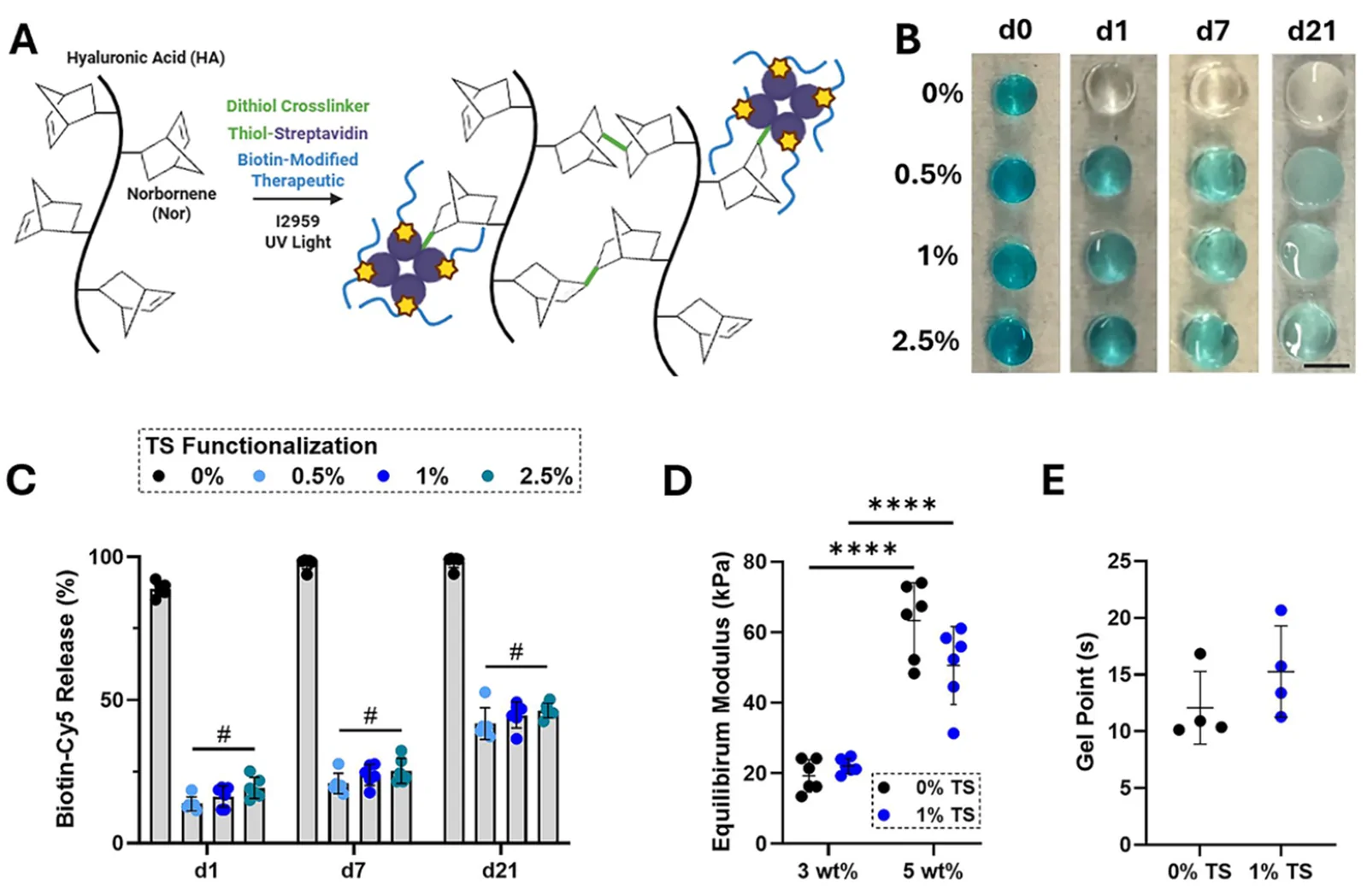
Enhancing Fluorophore Retention via Streptavidin Functionalization A) Schematic representation of hydrogel functionalization via reaction between thiol-streptavidin (TS) and the norbornene residues on the HA backbone. In the presence of biotin-modified therapeutics (e.g., nanocarriers, peptides, other biologics), a strong non-covalent bond forms with the streptavidin tetramer to localize the therapeutic. B) Representative hydrogel images (Scale = 5 mm) and C) percent release of 30 μM biotin-modified Cy5 encapsulated in hydrogels (3 wt%) with varying levels of TS (0, 0.5, 1, and 2.5%) after 1, 7 and 21 days of washing under pseudo-physiologic conditions (n=6 hydrogels/group). Release from 0.5, 1, and 2.5% TS hydrogels was statistically different than release from 0% TS hydrogels at every timepoint (Indicated by #; Two-Way Repeated Measures ANOVA with Tukey’s Multiple Comparisons). D) Equilibrium modulus of 3 and 5 wt% hydrogels (n=6 hydrogels/group) with and without TS (Two-Way ANOVA with Sidak’s Multiple Comparisons; ****p<0.0001). E) Gel point (intersection of storage and loss modulus during gelation) of 3 wt% hydrogels with 0% or 1% TS functionalization (n=4 hydrogels/group) (Mann-Whitney non-parametric t-test).

**Fig. 2.**
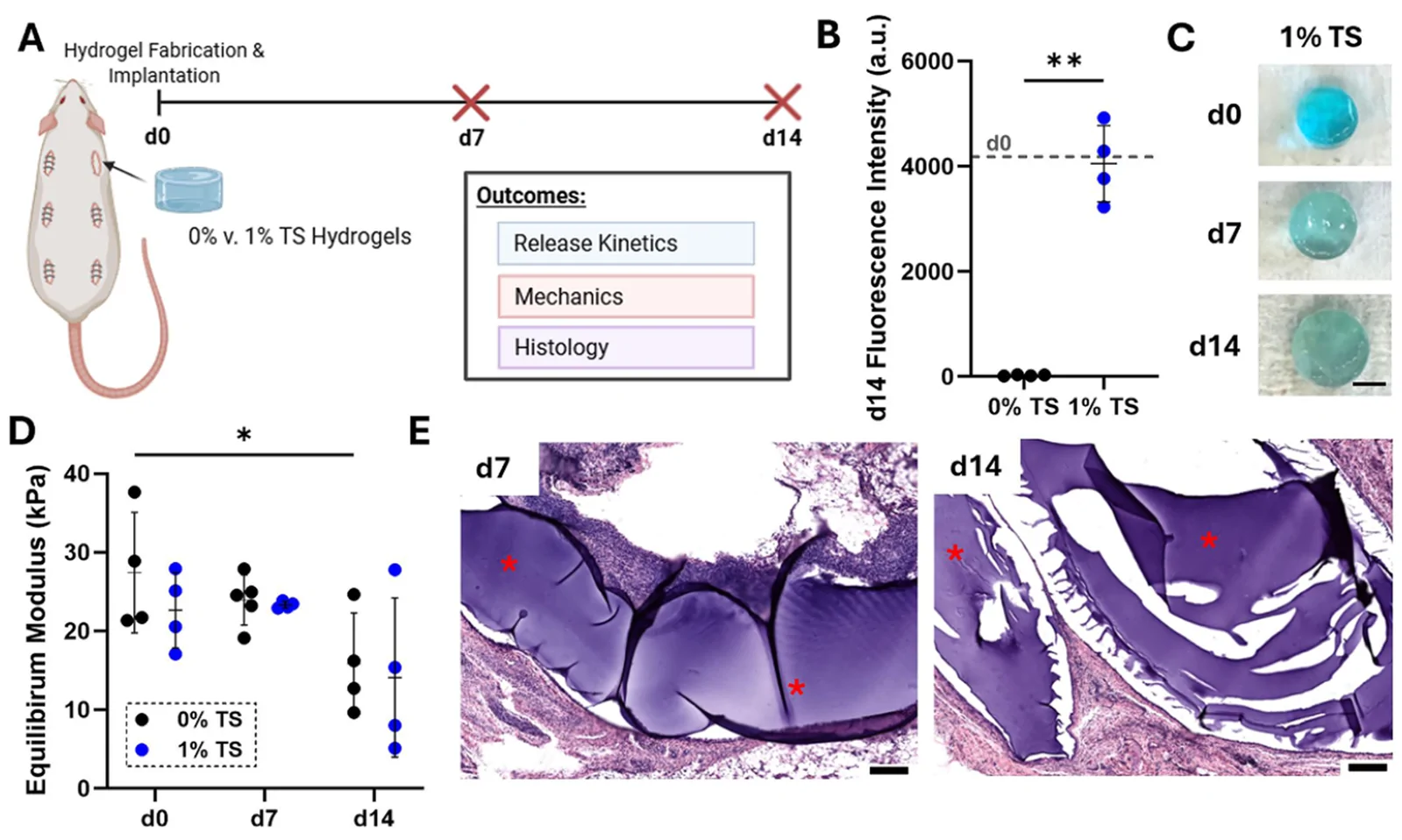
In Vivo Assessment of Streptavidin Functionalized Hydrogels A) Study design of subcutaneous implantation of hydrogels with and without TS functionalization for 7 and 14 days to assess the impact of functionalization on fluorophore release, mechanics, and tissue interactions (n=4 hydrogels/group for all outcomes). B) Fluorescence intensity of remaining biotin-modified fluorophore in 0% and 1% TS functionalized hydrogels after 14 days of subcutaneous implantation (Unpaired, parametric t-test with Welch’s Correction; **p<0.01). The dashed grey line represents the average fluorescence intensity of pre-implanted (d0) 0% and 1% TS hydrogels (~4150 a.u.). There were no significant differences between 1% TS and d0 controls (Unpaired, parametric t-test with Welch’s Correction). C) Representative images of hydrogels pre-implantation (d0) and after 7 and 14 days of implantation. Scale = 2.5 mm. D) Equilibrium moduli pre-implantation (d0) and after 7 and 14 days of implantation (Two-Way ANOVA with Sidak’s Multiple Comparison; *p<0.05). E) Representative H&E histology of 1% TS hydrogels implanted subcutaneously for 7 and 14 days showing minimal cell infiltration. The hydrogel is indicated by red asterisks. Scale= 250 μm.

**Fig. 3.**
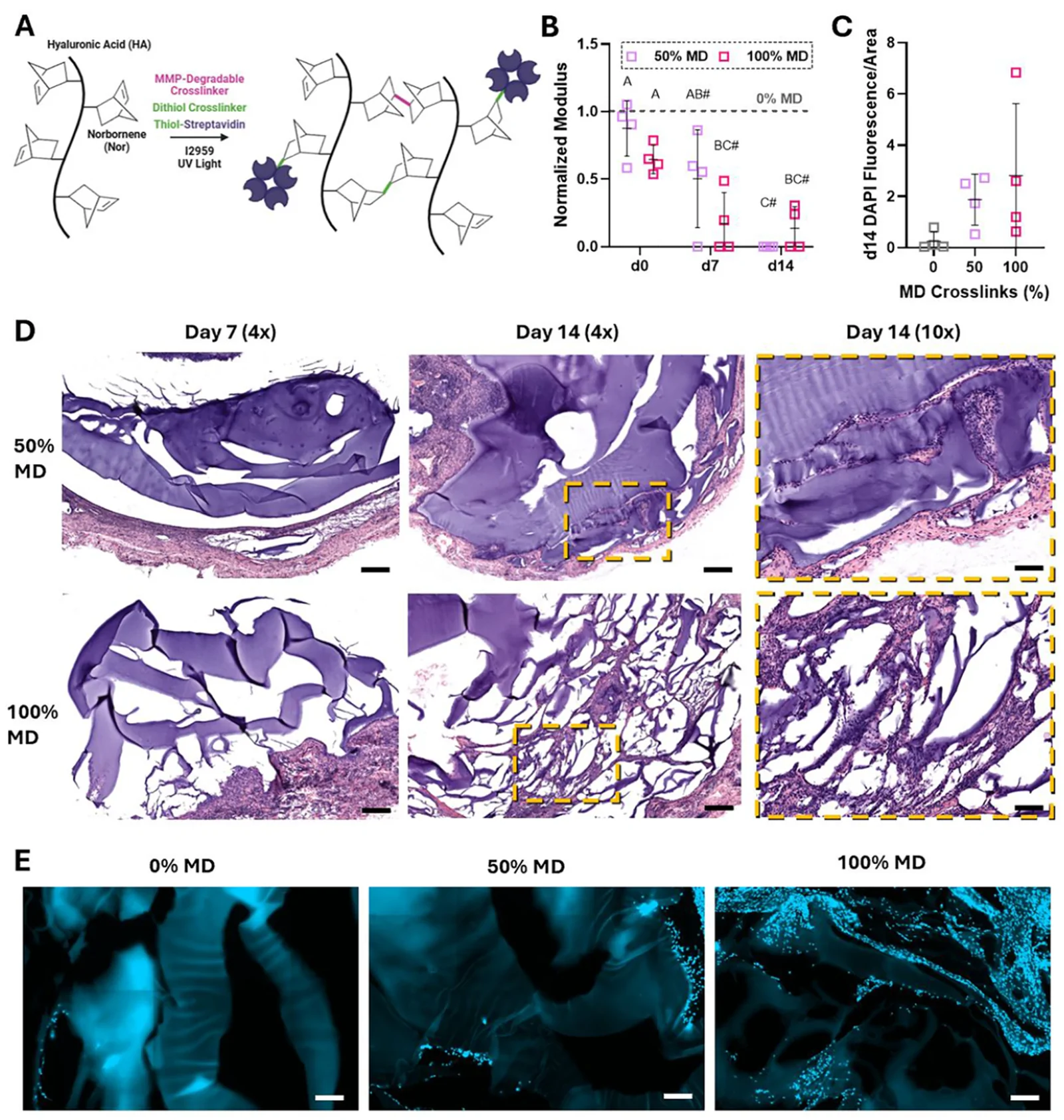
Introduction of MMP-Degradable Crosslinks Improves Cellular Infiltration A) Schematic representation of the introduction of both non-degradable and MMP-degradable (MD) crosslinks into hydrogels via reaction with norbornene residues on the HA (n=4 hydrogels/group). B) Equilibrium moduli of 50% and 100% MD hydrogels with 1% TS functionalization were normalized to pre-implanted 0% MD, 1% hydrogels, represented by the grey dashed line (Two-Way ANOVA with Sidak’s Multiple Comparisons). Different letters indicate significant statistical difference of p≤0.05. # indicates significant statistical difference from 0% MD hydrogels at each timepoint (One-Way ANOVA with Tukey’s Multiple Comparisons). Samples that were too degraded to mechanically test were recorded as having a modulus of zero. C) Cell infiltration into 1% TS hydrogels after 14 days of implantation as measured by DAPI fluorescence intensity within the fibrous capsule (Kruskal-Wallis Test;*p=0.0482 for the factor of MD). D) Representative H&E histology of 1% TS hydrogels with 50% and 100% MD crosslinks implanted subcutaneously for 7 and 14 days. Scale = 250 μm. Images were zoomed in to highlight nascent tissue deposition as cells infiltrated into the hydrogel. Scale = 100 μm. E) Representative DAPI images of 1% TS hydrogels with 0%, 50%, and 100% MD crosslinks after 14 days of implantations. Scale =250 μm.

**Fig. 4.**
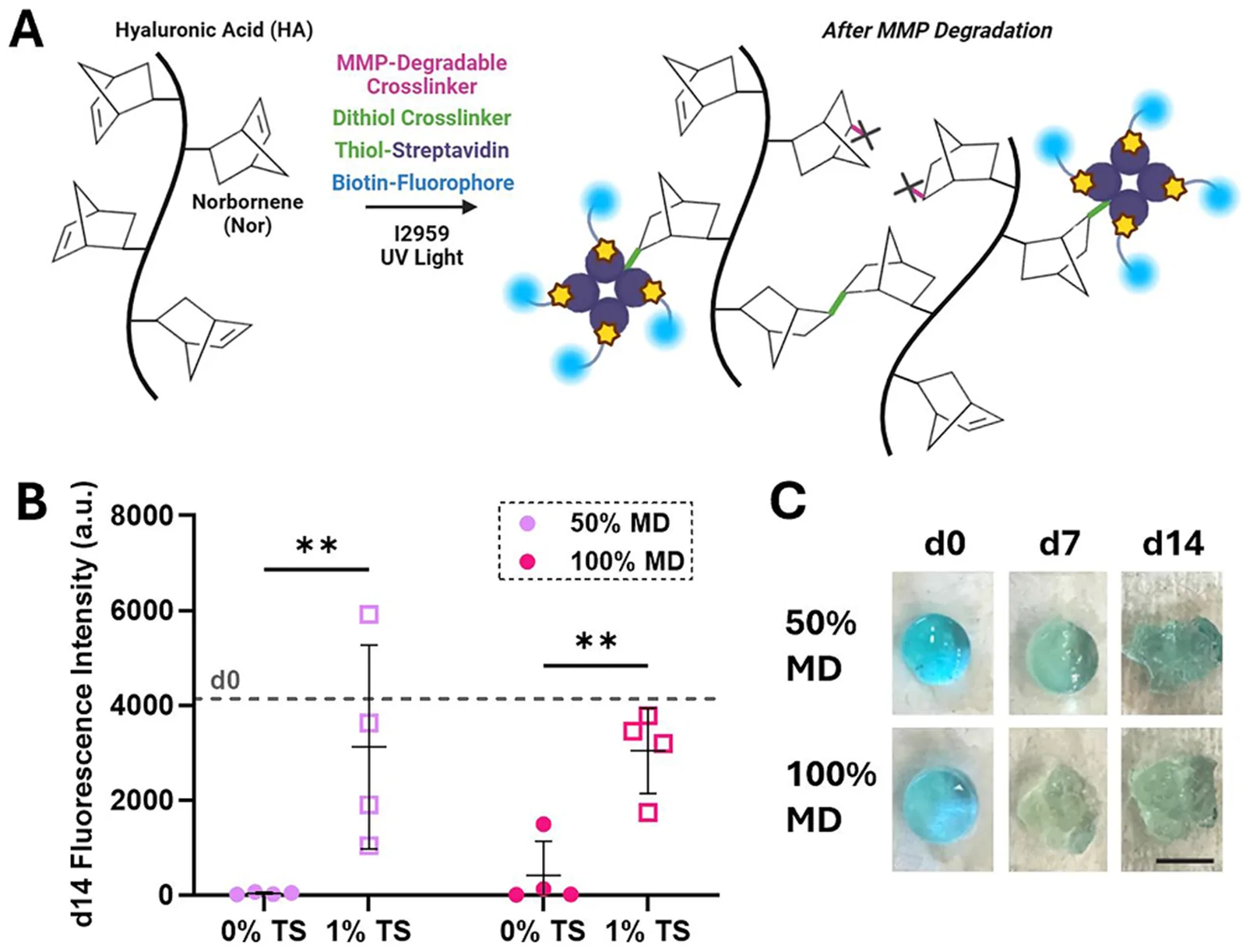
TS Hydrogels Retain Fluorescence Despite Degradation A) Schematic representation of the degradation of MD crosslinks and the retention of a biotin-modified fluorophore despite this degradation. B) Fluorescence intensity of 0% and 1% functionalized TS hydrogels (n=4 hydrogels/group) with 50% or 100% MD crosslinks after 14 days of subcutaneous implantation (Two-Way ANOVA with Sidak’s Multiple Comparisons; **p<0.01). The dashed grey line represents the average fluorescence intensity of pre-implanted (d0) 0% and 1% TS hydrogels (~4150 a.u.). There was no significant difference between 1% TS and d0 controls for both hydrogel groups (One-Way ANOVA with Tukey’s Multiple Comparisons). C) Representative hydrogel images at each timepoint. Scale = 5 mm.

**Fig. 5.**
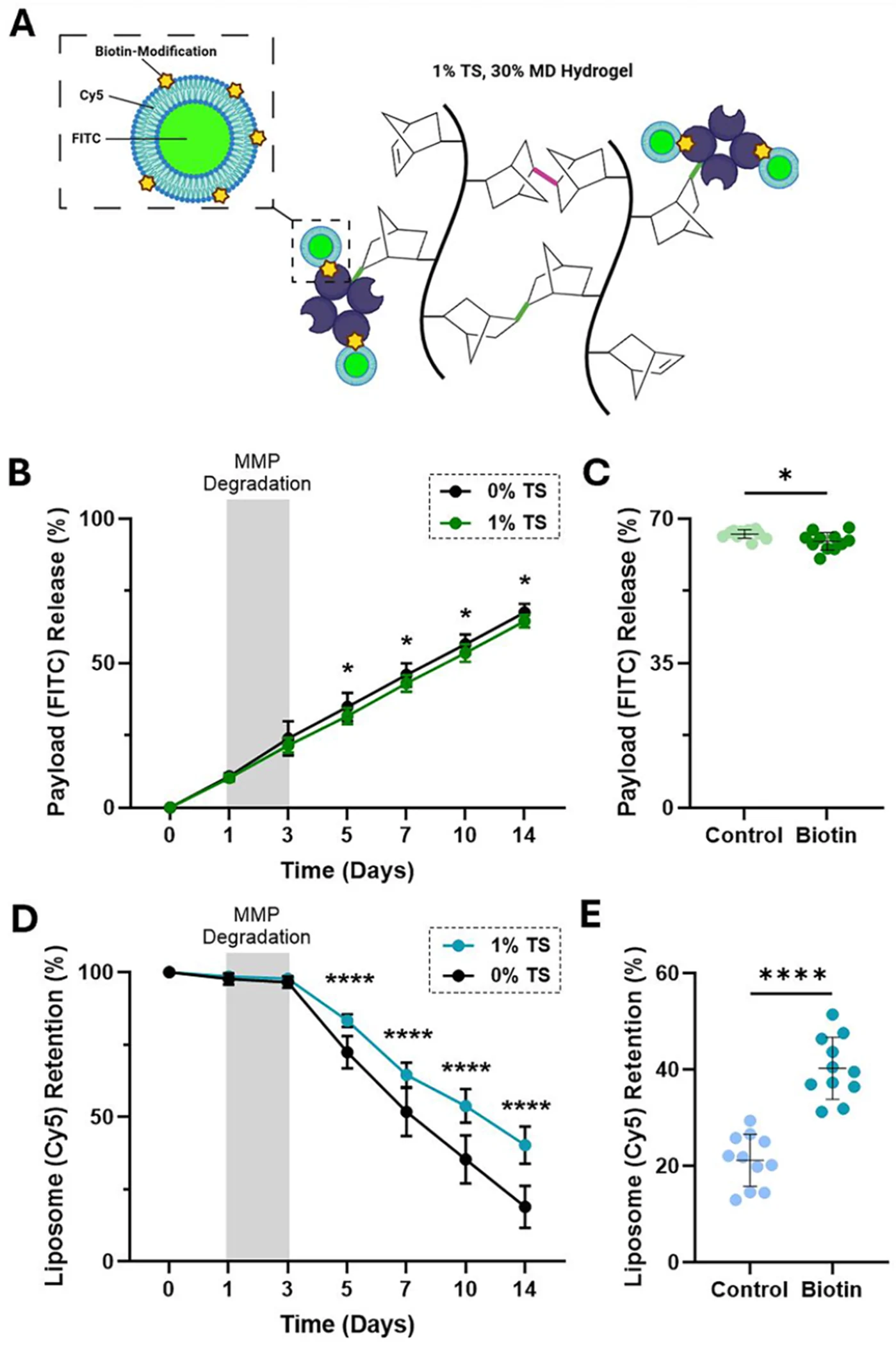
Nanocarrier Delivery via TS Hydrogels A) Schematic representation of the biotin-modified liposomes interacting with TS functionalized hydrogels. Liposomes contained Cy5 in the outer shell and FITC in inner core. The MD crosslinks were digested with collagenase during days 1–3 of washing to increase hydrogel mesh size. B) Release of FITC from biotin-modified liposomes from 0% and 1% TS functionalized hydrogels (n=11 hydrogels/group) over 14 days of pseudo-physiologic conditions (Two-Way ANOVA with Tukey’s Multiple Comparisons; *p<0.05). C) Total FITC release from liposomes without (Control) and with biotin-modification (Unpaired, parametric t-test with Welch’s Correction; *p<0.05). D) Retention of biotin-modified liposomes in 0% and 1% functionalized TS hydrogels with 30 % MD crosslinks (n=11 hydrogels/group) over 14 days of pseudo-physiologic conditions (Two-Way ANOVA with Tukey’s Multiple Comparisons; ****p<0.0001). E) Total retention of liposomes without (Control) and with biotin-modification after 14 days (Unpaired, parametric t-test with Welch’s Correction; ****p<0.0001).
