# Supplementary material for: Streptavidin functionalized hyaluronic acid hydrogels for controlled and customizable drug delivery
Source: Acta Biomater. Author manuscript; Available in PMC 2026 Oct 28. (PMC13715564; doi:10.1016/j.actbio.2026.07.015)
Supplement: Supplementary file 1 [file NIHMS2216445-supplement-supplemental_materials.docx]

**Appendix**

**Table S.1:** Exact hydrogel formulations used in this study (to fabricate n=6 hydrogels per group; 50 µL per hydrogel). Stock concentrations: NorHA (20% Modification), TS (2.8 tetramers per thiol, 50 mg/mL), DTT (20 mg/mL), MD (50 mg/mL), and Irgacure (5 mg/mL). For secondary reaction validation experiments, 1.8 µL of biotin-modified Cy5 (5 mM) was added in place of PBS to reach a hydrogel concentration of 30 µM. For implantation studies, 6 µL of RGD peptide (50 mg/mL) was added in place of PBS.

|  | **NorHA (mg)** | **TS (µL)** | **DTT (µL)** | **MD (µL)** | **Irg. (µL)** | **PBS (µL)** | **Figures** |
| --- | --- | --- | --- | --- | --- | --- | --- |
| **3 wt% TS0** |  |  |  |  |  |  |  |
| 0% MD | 9 | - | 8.63 | - | 3 | 273 | Fig. 2, 3 |
| 30% MD | 9 | - | 3.45 | 20.90 | 3 | 264 | Fig. 5 |
| 50% MD | 9 | - | 4.31 | 17.42 | 3 | 260 | Fig. 3, 4 |
| 100% MD | 9 | - | - | 34.83 | 3 | 247 | Fig. 3, 4 |
| **3 wt% TS1** |  |  |  |  |  |  |  |
| 0% MD | 9 | 19.18 | 8.63 | - | 3 | 260 | Fig. 2, 3 |
| 30% MD | 9 | 19.18 | 3.45 | 20.90 | 3 | 245 | Fig. 5 |
| 50% MD | 9 | 19.18 | 4.31 | 17.42 | 3 | 247 | Fig. 3, 4 |
| 100% MD | 9 | 19.18 | - | 34.83 | 3 | 234 | Fig. 3, 4 |
| **5 wt% TS0** |  |  |  |  |  |  |  |
| 0% MD | 15 | - | 14.38 | - | 3 | 268 | Fig. 1D |
| **5 wt% TS1** |  |  |  |  |  |  |  |
| 0% MD | 15 | 31.96 | 14.38 | - | 3 | 236 | Fig. 1D |

**Table S.2:** Moles of TS per 50 µL hydrogel for each level of functionalization investigated in this study. For experiments monitoring the release of a biotin-modified fluorophore, 1.5 E -9 moles of biotin-Cy5 was encapsulated per hydrogel.

| **Percent TS Functionalization (%)** | **Moles of TS** |
| --- | --- |
| 0.5% | 1.5 E -10 |
| 1% | 2.9 E -9 |
| 2.5% | 7.3 E -9 |

**Figure S.1: ^1^H NMR Characterization of Norbornene-Modified HA** A) Norbornene modification of ~20% was determined by integration of the vinyl peaks (2H, highlighted in green) relative to the methyl group of HA (3H, highlighted in purple).

**Figure S.2: Secondary Reaction Validation** A) Percent release of a biotin-modified fluorophore from hydrogels functionalized with varying levels of TS (n=6 hydrogels/group). Hydrogels with TS have a significantly lower percent release compared to 0% TS hydrogels at every timepoint (Two-Way Repeated Measures ANOVA with Tukey’s Multiple Comparisons; ****p<0.0001). There were no significant differences in percent release from 0.5%, 1%, and 2.5% TS hydrogels at any timepoint. B) Raw fluorescence intensity values of the media collected from 0.5%, 1%, and 2.5% TS hydrogels at each timepoint. While shown differently, this plot demonstrates the same information as Fig. S2A, which is that there is an initial release of unbound fluorophore after overnight incubation followed by gradual, slow release of the fluorophore from TS hydrogels. 2.5% TS hydrogels had a significantly higher fluorescence intensity compared to 0.5% TS hydrogels at day 1 and 0.5% and 1% TS hydrogels on day 7 (Two-Way Repeated Measures ANOVA with Tukey’s Multiple Comparisons; *p<0.05).

**Figure S.3: 6 Month Hydrogel Mechanics & Fluorophore Attachment** A) Equilibrium modulus of 5 wt% hydrogels with 0% and 1% TS immediately after fabrication and 6 months of storage at 4 ℃ (Two-Way Repeated Measure ANOVA with Multiple Comparisons; n= 4 hydrogels/group). B) Retention/release of a biotin-modified fluorophore at 0, 1, and 7 days after 1 hour incubation in 30 µM biotin-modified Cy5 (Scale = 2mm).

**Figure S.4: Rheologic Characterization** Average storage (G’) and loss (G’’) modulus on a log-log scale for A) 0% TS and B) 1% TS hydrogels (n=4 hydrogels/group). C) Storage modulus during gelation of 0% and 1% TS hydrogels during UV crosslinking. UV light exposure began after 45 seconds and lasted for approximately 2 minutes (denoted by grey box). D) Storage modulus after gelation (Unpaired t-test with Welch’s Correction). There were no significant differences in gelation between functionalized and non-functionalized hydrogels.

**Figure S.5: In Vitro Validation of MMP-Degradable Crosslinks** A) Swelling ratio 0% TS (closed symbol) and 1% TS (open symbol) hydrogels immediately after fabrication (circles) and after MMP degradation with 50 U/mL collagenase (squares) (Two-Way ANOVA with Tukey’s Multiple Comparisons; *p<0.05, ****p<0.0001; n=3 hydrogels/group). B) Percent release of biotin-modified Cy5 from 1% TS hydrogels with varying levels of MD crosslinks (Two-Way ANOVA with Tukey’s Multiple Comparisons; *p<0.05; # indicates significance p≤0.05 from all other groups at that timepoint). After media collection on d0, the washing media was replaced with 50 U/mL collagenase for two days to degrade the MD crosslinks. Media was collected and replaced with fresh PBS for three days. After media collection on d4, the washing media was replaced with 100 U/mL hyaluronidase to fully digest any remaining hydrogel.

**Figure S.6: MMP Degradable Hydrogel Mechanics** Equilibrium modulus of hydrogels with 0%, 50%, and 100% MD crosslinks immediately after fabrication for 0% TS (circles) and 1% TS (squares) hydrogels. These samples served as non-implanted (d0) controls for the subcutaneous implantation experiments (n=4 hydrogels/group). The inclusion of MD crosslinks significantly decreased modulus in 0% TS hydrogels but not 1% TS hydrogels (One-Way ANOVA with Tukey’s Multiple Comparisons; *p<0.05).

**Figure S.7: Normalized Hydrogel Mechanics** A) Equilibrium moduli of 50% and 100% MD hydrogels with 0% TS functionalization were normalized to pre-implanted 0% MD, 0% hydrogels, represented by the grey dashed line (Two-Way ANOVA with Sidak’s Multiple Comparisons; n=4 hydrogels/group). There were no significant differences between MD hydrogels at any timepoint. # indicates significant statistical difference from 0% MD hydrogels at each timepoint (One-Way ANOVA with Tukey’s Multiple Comparisons). B-D) Individual comparisons of normalized modulus at each timepoint (Two-Way ANOVA with Sidak’s Multiple Comparisons; *p<0.05), with significant differences only observed between 0% and 1% TS hydrogels with 50% MD crosslinks at d0 (pre-implantation).

**Figure S.8: Day 7 Histology** H&E histology for 0% and 1% TS functionalized hydrogels with varying levels of MD crosslinks (0%, 50%, and 100%) after 7 days of subcutaneous implantation. Each column represents a biologic replicate (n=4). 4x magnification. Scale = 100 µm.

**Figure S.9: Day 14 Histology** H&E histology for 0% and 1% TS functionalized hydrogels with varying levels of MD crosslinks (0%, 50%, and 100%) after 14 days of subcutaneous implantation. Each column represents a biologic replicate (n=4). 4x magnification. Scale = 100 µm.

**Figure S.10: Cell Infiltration** Representative images of 0% and 1% TS hydrogels with 0%, 50%, and 100% MD crosslinks after 7 and 14 days of implantation stained with DAPI to identify cells infiltrating into the hydrogel. Scale = 100 µm.

**Figure S.11: Additional Data from Subcutaneous Implantation Study** Individual comparisons (Two-Way ANOVA with Sidak’s Multiple Comparisons) of and cell infiltration at A) d7 and B) d14 for 0% and 1% TS hydrogels with varying levels of MD crosslinks (n=4 hydrogels/group). C) Combined analysis of cell infiltration at d14 (Kruskal Wallis with Multiple Comparisons; *p<0.05). D) Fluorescence intensity of hydrogels implanted subcutaneously for 7 days (Two-Way ANOVA with Sidak’s Multiple Comparisons; **p<0.01; n=4 hydrogels/group). Two samples in the 0% TS, 0% MD hydrogel group were unrecoverable. When comparing 1% TS hydrogels, 50% and 100% MD hydrogels had significantly lower fluorescence intensity than 0% MD hydrogels (statistics not shown). The grey dashed line represents the average fluorescence intensity of pre-implanted (d0) controls (~4,150). 0% and 100% MD hydrogels with 1% TS functionalization had statistically different intensity values than non-implanted controls (One-Way ANOVA with Tukey’s Multiple Comparisons). Void area (i.e., space without hydrogel within the fibrous capsule) after E) 7 days and F) 14 days (One-Way ANOVA with Tukey’s Multiple Comparisons; n=4 hydrogels/group).
